# Analysis of mold and mycotoxins in naturally infested indoor building materials

**DOI:** 10.1007/s12550-022-00461-3

**Published:** 2022-07-28

**Authors:** Viktoria Lindemann, Tim Schleiner, Ulrich Maier, Hubert Fels, Benedikt Cramer, Hans-Ulrich Humpf

**Affiliations:** 1 Institute of Food Chemistry, Westfälische Wilhelms-Universität Münster, Corrensstr. 45, 48149 Münster, Germany; 2 Umweltlabor ACB GmbH Münster, Albrecht-Thaer-Straße 14, 48147 Münster, Germany

**Keywords:** Mold, *Stachybotrys*, Mycotoxins, Indoor, Building materials

## Abstract

Health issues of residents of mold-infested housing are reported on a regular basis, and reasons for the arising impairments can be manifold. One possible cause are the toxic secondary metabolite produced by indoor microfungi (mycotoxins). To enable a more thorough characterization of the exposure to mycotoxins in indoor environments, data on occurrence and quantities of mycotoxins is essential. In the presented study, 51 naturally mold-infested building material samples were analyzed applying a previously developed method based on ultra-high performance liquid chromatography (UHPLC) separation in combination with triple-quadrupole mass spectrometry (TQMS) detection. A total of 38 secondary metabolites derived from different indoor mold genera like *Aspergillus*, *Fusarium*, *Penicillium*, and *Stachybotrys* were analyzed, of which 16 were detectable in 28 samples. As both the spectrum of target analytes and the investigated sample matrices showed high chemical varieties, an alternative calibration approach was applied complementary to identify potentially emerging matrix effects during ionization and mass spectrometric detection. Overall, strong alterations of analyte signals were rare, and compensation of considerable matrix suppression/enhancement only had to be performed for certain samples. Besides mycotoxin determination and quantification, the presence of 18 different mold species was confirmed applying microbiological approaches in combination with macro- and microscopic identification according to DIN ISO 16000–17:2010–06. These results additionally highlight the diversity of mycotoxins potentially arising in indoor environments and leads to the assumption that indoor mycotoxin exposure stays an emerging topic of research, which has only just commenced.

**Supplementary Information:**

The online version contains supplementary material available at 10.1007/s12550-022-00461-3.

## Introduction

Mycotoxins are a diverse group of chemical compounds formed by the secondary metabolism of microfungi with harmful effects to vertebrates (Bennett and Klich 2003). As molds are ubiquitously distributed in the environment and the presence of certain species has even been documented on the International Space Station (Vesper et al. 2008), the same may be assumed for mycotoxins. Modern research concerning this topic was initiated about 60 years ago with the discovery of aflatoxins in the food and feed chain (Kensler et al. 2011). Today, fungi contaminating foodstuffs are still a global burden, affecting developing countries much more than developed regions such as the EU, where strict regulations are ensuring food safety (EC 2006; Schmidt et al. 2021a). However, other potential sources of exposure for humans to fungi and their secondary metabolites are indoor environments.

The most critical factor for indoor mold infestation is humidity caused by wrong ventilation habits or occurring after floodings (Górny et al. 2002; WHO 2009). Fungal contamination can be perceived either visually or through emerging odors caused by microbial volatile organic compounds (MVOCs). Measurements of MVOCs are performed on a regular basis by mold consultants, and emission profiles are regarded to be suitable for verifying the presence of certain, but not all filamentous fungi (Betancourt et al. 2013; Schleibinger et al. 2005). In order to provide a secure identification and characterization of indoor mold exposure, directly contaminated building materials, swab samples, and sampling of air on different media (object slides, petri dishes with filter material/agar) with subsequent microscopic or morphologic differentiation are more reliable alternatives applied in routine analysis (DIN ISO 16000–17:^2010^–06). An assessment of toxicological concern, however, is not provided by these approaches.

Similar to the consumption of contaminated food, the exposure to mold in residential settings is considered to be critical for human health as it may cause or promote allergic reactions and fungal infections (Baxi et al. 2016; Robbins et al. 2000). Moreover, medical documentations of people showing diseases, especially of the respiratory tract, and further symptoms of intoxications are manifold (Brewer et al. 2013; Hooper et al. 2009; Thrasher et al. 2016; Johanning et al. 1999). In exceptional cases, more extreme courses of illness were reported, leading to deaths at worst (Croft et al. 1986; Dearborn et al. 1999). However, the correlation between indoor mold exposure and health impairments is still scientifically controversial, and it was not yet possible to identify a potentially exclusive trigger for emerging diseases. It is known that both spores and fragments of mycelium can be released into the air (Górny et al. 2002), and they have both been associated with adverse health effects after inhalation (Baxi et al. 2016; Górny 2004; WHO 2009). Additionally, the inhalative uptake of mycotoxins is suspected to pose higher toxic effects (Creasia et al. 1990) and to induce deviating toxicity (Jakšić et al. 2020). However, not all homes of affected inhabitants show high levels of airborne fungal components indicating the presence of additional agents responsible for health impairments.

The part of mycotoxins in the illustrated medical complications, which are summarized in the term “sick building syndrome” (SBS) (Mahmoudi and Gershwin 2009), is a recurring topic of discussion among consultants and medical and scientific experts. Certain mycotoxins are known to have the potential to cause typical but unspecific symptoms of SBS including headache, fatigue, nausea, and irritations of the respiratory tract in humans. The determination of mycotoxins indoors is however currently not performed during routine analyses but has been a regular topic of research in the last decades. Compared to mycotoxin analysis in food and feed, some different factors are worth being considered in the residential setting: The expectations towards occurring secondary metabolites must be adapted as other microfungi like *Acremonium*, *Cladosporium*, and *Stachybotrys* species show higher prevalence in indoor environments (Andersen et al. 2011; Hyvärinen et al. 2002; Reboux et al. 2009). Furthermore, in comparison to mycotoxins in food, inhabitants of mold affected housing can ingest mycotoxins in different ways depending on the contaminated medium. In the past, mycotoxin analysis was predominantly performed on directly mold-infested building materials (e.g., wallpaper, gypsum board), in house dust, and after sampling of air. A clear focus was set on toxins derived from *Stachybotrys* as this specific genus is often made accountable for symptoms of SBS (Assouline-Dayan et al. 2002; Eppley and Bailey 1973; Johanning et al. 1996). Especially macrocyclic trichothecenes (MCTs) have been included in the majority of studies as they are highly toxic and can inhibit protein biosynthesis (Jarvis 2003; Nielsen et al. 2009).

Generally, highest indoor mycotoxin levels were determined on material samples, and, as mentioned before, in particular the prevalence of MCTs is well documented (Gottschalk et al. 2006; Jagels et al. 2020; Nikulin et al. 1994; Tuomi and Reijula 1998) with quantities ranging up to 12 µg/cm^2^ on naturally infested wallpaper (Gottschalk et al. 2006). Besides MCTs, Jagels et al. were able to quantify high levels of mycotoxins belonging to another group of secondary metabolites produced by all *Stachybotrys* species: The phenylspirodrimanes (PSDs) (Jagels et al. 2019, 2020; Jarvis et al. 1995). Further toxic fungal compounds, whose presence was verified on indoor building materials, include *Alternaria*, *Aspergillus*, and *Penicillium* toxins such as alternariol (AOH), sterigmatocystin (STG), and gliotoxin (GTX) (Bloom et al. 2007, 2009b; Vishwanath et al. 2009). Besides investigations of naturally infested building materials, additional experiments were carried out following artificially induced mold growth in order to characterize the potential of various mold species to produce mycotoxins in indoor environments (Aleksic et al. 2016; Jagels et al. 2020; Nielsen et al. 1999). Overall, the performed analyses give first indications on potentially relevant compounds, but the information gained for a reliable exposure assessment is limited, as a direct uptake, besides in dermal form, from the described matrices is unlikely. However, mycotoxins are partially transferred to other indoor media like air and dust alongside spores or fungal fragments. In house dust, mycotoxin levels are lower, but the potential of human intake is elevated as oral and dermal uptake of present mycotoxins is enabled (Butte and Heinzow 2002). Studies investigating mycotoxins in house dust revealed, among others, the presence of several known secondary metabolites of *Aspergillus*, *Fusarium*, *Penicillium*, and *Stachybotrys* (Bloom et al. 2009a; Došen et al. 2016; Lindemann et al. 2022; Richard et al. 1999; Vishwanath et al. 2011). Furthermore, as in particular, the incidence of diseases of the respiratory tract is increased after indoor mold exposure, the analysis of mycotoxins in air is of special interest. An inhalation of mycotoxins is possible as part of airborne particles (Brasel et al. 2005) or after aerosolization of guttation fluids (Gareis et al. 2014; Salo et al. 2019). Comparatively few studies, again focusing on MCTs in air samples (Brasel et al. 2005; Gottschalk et al. 2008), have been conducted in residential settings, even though mycotoxins are known to be hazardous air contaminants (Jarvis and Miller 2005; Miller and McMullin 2014).

The route of intake of mycotoxins immensely affects their toxicity and bioavailability and ultimately defines their responsibilities for health impairments of inhabitants of mold-infested housing. According to the WHO, mycotoxins in indoor environments should be classified as potential health hazards, even though there is no strong evidence relating indoor mycotoxin exposure to arising diseases (WHO 2009). Therefore, there is a definite need to identify potential mechanisms of action to subsequently elucidate potentially occurring symptoms. Moreover, further studies of occurring mycotoxins derived from various mold species, which are consequently worth to be considered in these toxicity studies, need to be performed.

The presented study was conducted to provide further data on the occurrence and quantities of mycotoxins in indoor environments. It describes the identification and quantification of a broad variety of mycotoxins derived from several mold genera such as *Aspergillus*, *Fusarium*, *Penicillium*, and *Stachybotrys* in naturally infested indoor building materials by ultra-high performance liquid chromatographic separation coupled to triple-quadrupole mass spectrometric detection (UHPLC-TQMS). The detection method included 38 target analytes, and an authentic set of 51 naturally mold-infested samples was analyzed. Due to a large spectrum of various building materials and sample matrices, matrix-matched calibration was not applicable, and quantification was carried out by solvent calibration. Samples showing quantifiable mycotoxin levels were additionally analyzed by the echo-peak technique, which enabled the estimation of matrix effects of the diverse building materials (Zrostlíková et al. 2002). Further characterization of the samples was carried out applying microbiological approaches in combination with macro- and microscopic identification of present mold genera and classification of the extent of contamination based on colony-forming unit (CFU) counts.

## Materials and methods

### Chemicals and reagents

A Purelab Flex 2 system (Veolia Water Technologies, Celle, Germany) was used for water purification (ASTM type 1 grade). Acetonitrile (MeCN) in LC–MS-grade purity was obtained from Fisher Scientific (Schwerte, Germany), and formic acid (FA) was purchased from Merck KGaA (Darmstadt, Germany). The origin of the mycotoxin standard substances has been described in a previous publication (Lindemann et al. 2022). The names, abbreviations, and structures of all considered mycotoxins are listed in Table S1 of the Supplementary Information. All analytes were combined in one working solution for TQMS analysis at 100-fold concentration of the highest calibration point. The solution was stored at − 18 °C.

Chemicals utilized during the experiments on mold differentiation as well as during the determination of CFUs included sodium chloride (AppliChem GmbH, Darmstadt, Germany), buffered sodium chloride peptone solution (Merck KGaA, Darmstadt, Germany, + 0.01% Tween 80, AppliChem GmbH, Darmstadt, Germany), dichloran glycerin agar containing chloramphenicol (DG18 agar, Thermo Fisher Scientific, Wesel, Germany), and malt extract agar containing chloramphenicol (MEA, Thermo Fisher Scientific, Wesel, Germany).

### Sample collection

Subsamples (*n* = 51) of building materials were taken in indoor housing (24 households) in the north-west of Germany. Materials included directly mold-infested wallpapers (*n* = 5), plasters (*n* = 2), wood (*n* = 2), and different isolation materials like (styro-)foam (*n* = 35) and glass wool (*n* = 7). A detailed list of the investigated sample materials and origins is presented in Table 1.

**Table 1:** Information on the occurrence and material type of the samples investigated in the presented studies. Furthermore, according to DIN ISO 16000–17:^2010^–06 regulations, determined mold species as well as by the UHPLC-TQMS analysis detected mycotoxins are listed

Sample no	Household no	Material	Determined mold genera/species	CFU[/g]	Detectable mycotoxins
1	1	Plaster	*Penicillium* spp.	1.2 × 10^5^	n.d.
2		Wallpaper	*Penicillium* spp.	6.0 × 10^3^	ACDIAL AC, L-671, STCHR B, SAT G, SAT H, ST B, ST C, STAM, STBON D, STDIAL, STDIAL AC, STLAC, STLAC AC
			*Stachybotrys* sp.	8.0 × 10^6^	
3		Wallpaper	*Acremonium* sp.	3.0 × 10^5^	n.d.
			*Penicillium* spp.	2.3 × 10^6^	
			*Scopulariopsis* sp.	4.3 × 10^6^	
4		Wallpaper	*Aspergillus calidoustus*	8.0 × 10^3^	n.d.
			*Paecilomyces* sp.	3.0 × 10^2^	
			*Penicillium* spp.	7.0 × 10^3^	
5	2	Styrofoam	*Aspergillus versicolor* complex	2.1 × 10^6^	ACDIAL AC, L-671, STG, ST B, ST C, STAM, STBON D, STDIAL, STDIAL AC, STLAC, STLAC AC
			*Chaetomium* sp.	4.0 × 10^5^	
			*Penicillium* sp.	9.0 × 10^4^	
			*Stachybotrys* sp.	2.0 × 10^6^	
6		Styrofoam	*Aspergillus versicolor* complex	1.0 × 10^6^	ACDIAL AC, L-671, STG, ST B, ST C, STAM, STBON D, STDIAL, STDIAL AC, STLAC, STLAC AC
			*Chaetomium* sp.	1.3 × 10^6^	
			*Penicillium* sp.	7.0 × 10^4^	
			*Stachybotrys* sp.	1.0 × 10^5^	
7		Styrofoam	*Aspergillus versicolor* complex	2.0 × 10^6^	ACDIAL AC, L-671, STG, ST B, ST C, STAM, STBON D, STDIAL, STDIAL AC, STLAC, STLAC AC
			*Chaetomium* sp.	2.0 × 10^6^	
			*Penicillium* sp.	5.0 × 10^5^	
			*Stachybotrys* sp.	1.0 × 10^5^	
8		Styrofoam	*Aspergillus versicolor* complex	5.0 × 10^5^	ACDIAL AC, L-671, STG, ST B, ST C, STAM, STBON D, STDIAL, STDIAL AC, STLAC, STLAC AC
			*Chaetomium* sp.	2.0 × 10^6^	
			*Penicillium* sp.	2.0 × 10^5^	
			*Stachybotrys* sp.	1.0 × 10^5^	
9	3	Wallpaper, painted	*Aspergillus* section *Nigri*	3.0 × 10^4^	STG
			*Aspergillus versicolor* complex	7.0 × 10^5^	
			*Aureobasidium* spp.	1.0 × 10^5^	
			*Cladosporium* spp.	5.0 × 10^5^	
			*Penicillium* spp.	7.0 × 10^4^	
10	4	Chipboard	*Aspergillus versicolor* complex	1.0 × 10^4^	ENB, STG
			*Chrysonilia* sp.	1.0 × 10^3^	
			*Fusarium* sp.	1.0 × 10^4^	
			*Penicillium* spp.	1.2 × 10^5^	
			*Phoma* sp.	2.0 × 10^4^	
11		Glass wool	*Aspergillus versicolor* complex	1.0 × 10^3^	n.d.
			*Chrysonilia* sp.	2.0 × 10^2^	
			*Penicillium* spp.	4.0 × 10^2^	
			*Phoma* sp.	1.0 × 10^5^	
12		Chipboard	*Aspergillus versicolor* complex	9.0 × 10^2^	ENB, STG
			*Penicillium* spp.	3.2 × 10^3^	
13	5	Styrofoam	*Acremonium* sp.	4.0 × 10^2^	n.d.
			*Aspergillus versicolor* complex	1.2 × 10^3^	
			*Stachybotrys* sp.	2.0 × 10^2^	
14	5	Styrofoam	*Aspergillus versicolor* complex	1.0 × 10^2^	STG
			*Chaetomium* sp.	1.0 × 10^2^	
			*Stachybotrys* sp.	1.0 × 10^2^	
15	6	Debris, styrofoam	*Aspergillus versicolor* complex	5.0 × 10^4^	STG
			*Penicillium* spp.	4.0 × 10^4^	
16	7	Debris, styrofoam	*Aspergillus versicolor* complex	5.0 × 10^2^	STG
			*Penicillium* spp.	2.0 × 10^2^	
17		Debris, styrofoam	n.d	n.d	STG
18	8	Styrofoam	*Aspergillus versicolor* complex	1.5 × 10^4^	STG
			*Penicillium* sp.	2.1 × 10^3^	
19		Styrofoam	*Acremonium* sp.	2.0 × 10^4^	STG
			*Aspergillus versicolor* complex	2.3 × 10^4^	
			*Penicillium* sp.	1.6 × 10^4^	
20		Styrofoam	*Acremonium* sp.	2.2 × 10^3^	n.d.
			*Aspergillus versicolor* complex	2.8 × 10^4^	
			*Penicillium* sp.	5.0 × 10^3^	
21	9	Styrofoam	*Acremonium* spp.	4.0 × 10^5^	ACDIAL AC, L-671, STBON D, ST C, STDIAL
			*Aspergillus calidoustus*	2.0 × 10^2^	
			*Aspergillus versicolor* complex	1.6 × 10^3^	
			*Stachybotrys* sp.	2.0 × 10^4^	
			*Tritirachium oryzae*	3.0 × 10^4^	
22	10	Debris	*Aspergillus versicolor* complex	4.0 × 10^2^	n.d.
			*Penicillium* spp.	4.0 × 10^2^	
23		Styrofoam	*Aspergillus versicolor* complex	2.1 × 10^5^	n.d.
			*Penicillium* spp.	8.0 × 10^2^	
24		Styrofoam	*Aspergillus versicolor* complex	1.8 × 10^4^	ENB
			*Penicillium* spp.	3.0 × 10^2^	
25	11	Styrofoam, foam material	*Aspergillus versicolor* complex	8.0 × 10^2^	STG
			*Penicillium* spp.	1.9 × 10^4^	
26	12	Styrofoam	*Acremonium* sp.	4.6 × 10^4^	L-671, STBON D
			*Aspergillus versicolor* complex	3.0 × 10^2^	
			*Penicillium* spp.	2.4 × 10^3^	
27		Styrofoam	*Acremonium* sp.	2.8 × 10^3^	n.d.
			*Penicillium* spp.	9.0 × 10^2^	
28		Styrofoam	*Fusarium* sp.	1.3 × 10^3^	n.d.
			*Penicillium* spp.	1.4 × 10^4^	
29	13	Styrofoam	*Acremonium* sp.	2.0 × 10^3^	STG
			*Aspergillus versicolor* complex	6.0 × 10^4^	
			*Penicillium* sp.	1.5 × 10^4^	
30		Styrofoam	*Aspergillus versicolor* complex	7.0 × 10^4^	STG
			*Penicillium* sp.	2.0 × 10^4^	
31		Styrofoam	*Acremonium* sp.	1.5 × 10^5^	STG
			*Aspergillus versicolor* complex	5.0 × 10^3^	
			*Penicillium* sp.	1.8 × 10^3^	
32	14	Styrofoam	*Acremonium* sp.	2.4 × 10^3^	n.d.
			*Aspergillus versicolor* complex	2.0 × 10^3^	
			*Fusarium* sp.	8.0 × 10^2^	
			*Penicillium* spp.	2.0 × 10^3^	
33		Styrofoam	*Acremonium* sp.	1.4 × 10^4^	STG
			*Aspergillus versicolor* complex	6.0 × 10^3^	
			*Penicillium* spp.	2.2 × 10^4^	
34		Styrofoam	*Acremonium* sp.	1.0 × 10^3^	n.d.
			*Aspergillus versicolor* complex	1.0 × 10^3^	
			*Penicillium* spp.	9.2 × 10^3^	
35	15	Styrofoam, gray	n.d	n.d	n.d.
36	16	Wallpaper	n.d	n.d	STG
37	17	Glass wool	*Penicillium* spp.	1.6 × 10^5^	n.d.
38		Glass wool	*Aspergillus versicolor* complex	3.0 × 10^3^	n.d.
			*Penicillium* spp.	1.3 × 10^4^	
39		Glass wool	n.d	n.d	n.d.
40		Glass wool	*Aspergillus versicolor* complex	4.0 × 10^3^	n.d.
			*Penicillium* spp.	2.9 × 10^5^	
41		Glass wool	*Aspergillus versicolor* complex	2.0 × 10^3^	n.d.
			*Penicillium* spp.	6.0 × 10^4^	
42	18	Foam material	n.d	n.d	n.d.
43		Foam material	n.d	n.d	n.d.
44	19	Styrofoam	n.d	n.d	STG
45	20	Styrofoam	*Acrostalagmus luteoalbus*	1.0 × 10^5^	ENA_1_, ENB, STBON D, STG
			*Cladosporium* spp.	2.4 × 10^4^	
			*Penicillium* spp.	8.0 × 10^4^	
			*Phoma* sp.	1.0 × 10^5^	
46		Styrofoam	*Acrostalagmus luteoalbus*	2.0 × 10^4^	STG
			*Aspergillus versicolor* complex	2.5 × 10^5^	
			*Cladosporium* spp.	4.6 × 10^3^	
			*Penicillium* spp.	6.8 × 10^4^	
			*Scopulariopsis* sp.	2.0 × 10^3^	
47	22	Styrofoam	*Aspergillus versicolor* complex	1.0 × 10^2^	n.d.
			*Penicillium* spp.	4.0 × 10^2^	
48	18	Styrofoam, colored	*Aspergillus versicolor* complex	2.4 × 10^3^	n.d.
			*Penicillium* spp.	6.0 × 10^2^	
49	23	Styrofoam	*Aspergillus versicolor* complex	1.5 × 10^3^	STG
			*Penicillium* spp.	1.1 × 10^3^	
			*Trichoderma* sp.	1.0 × 10^3^	
50		Styrofoam	*Aspergillus versicolor* complex	1.0 × 10^2^	n.d.
			*Penicillium* spp.	5.8 × 10^3^	
			*Trichoderma* sp.	1.3 × 10^4^	
51	24	Glass wool	*Aspergillus fumigatus*	1.0 × 10^2^	ENB
			*Aspergillus section Nigri*	1.0 × 10^2^	
			*Aspergillus versicolor* complex	4.5 × 10^4^	
			*Cladosporium* sp.	2.0 × 10^5^	
			*Penicillium* sp.	1.2 × 10^4^	

*n.d*. Not Detectable

### Detection and characterization of culturable mold in material samples

Experiments on microscopic and morphologic differentiation of mold genera/species as well as on the estimation of the extent of mold exposure were performed at the Umweltlabor ACB GmbH according to regulations of the German Institute for Standardization (DIN ISO 16000–17:^2010^–06).

A buffered sodium chloride peptone solution containing 0.01% Tween 80 was added to the comminuted material samples, usually at a ratio of 10:1. The suspensions were then mixed on a horizontal shaker for 15 min, and serial dilutions using 0.9% sodium chloride solution were prepared, resulting in a concentration range of 10^−2^ to 10^−5^ of the initial suspensions. One hundred microliters of each initial suspension and each dilution were then transferred to two DG18 plates and two MEA plates and distributed. One DG18 and MEA plate each was prepared in an analogue manner containing sterile sodium chloride solution and buffered sodium chloride peptone solution serving as negative controls.

For the detection of mesophilic molds, the DG18 plates and one of the MEA plates of each sample material were incubated at 22 °C for 3–7 days. The second MEA plates were incubated for the same time at 36 °C and were used for the determination of thermo-tolerant mold genera (e.g., *Aspergillus*).

Identification of the mold genera present in the analyzed samples was based on the morphology of the macroscopic and microscopic image applying common identification and differentiation literature (Baumgart 1994; BG BAU 2006; Klich 2002; Domsch et al. 2007; Guarro et al. 2012; Hoog and Guarro 1996; Petrini and Petrini 2010; Samson and Frisvad 2004; Samson and Houbraken 2010; Seifert et al. 2011; Umweltbundesamt 2017). A visual assignment to certain genera based on cultivar morphological characteristics was performed, depending on the expression of the growth, from the third to the seventh day of incubation. The macroscopic characteristics (growth type, color of the colony, noticeable features), mainly on MEA plates, were determined first. Afterwards, slides were prepared for light microscopic analyses (Zeiss Axio Lab A.1 microscope with 10 × ocular and 10, 50, 63, and 100 × objectives, Carl Zeiss, Oberkochen, Germany). If necessary, an aniline blue staining step was performed for 15–30 min applying a solution containing 0.25 g aniline blue (Waldeck GmbH & Co. KG, Münster, Germany) in 250 mL 80% lactic acid (Honeywell/Fluka, Seelze, Germany). Results of the analyses are presented in Table 1 (column “detectable molds”). Characterization was made at least at genus level, and molds known to be mycotoxin producers were differentiated down to the species level.

The colony count was performed visually (without the aid of a magnifying glass), taking all levels of dilution into account. For the calculation of the mold concentration, initially only the DG18 agar plates were used. On the MEA medium, only those mold genera were evaluated that do not grow or form spores on DG18 agar like *Chaetomium* and *Stachybotrys*. Agar plates showing least interferences between single colonies but enough colonies (10–100) for valid quantification were selected. The arithmetic mean was determined from the colony count ($\sum CFU$) and the number of plates (*n*) of the evaluable dilutions (*d*) and expressed as CFU per gram:

$$CFU/g= \frac{\sum CFU}{n} \cdot d$$

Determined CFUs of the analyzed samples are presented in Table 1.

### Sample preparation for analysis of mycotoxins

A 1-cm^2^ piece of each sample was cut out using spatulas and tweezers and transferred into a 50-mL screw cap polypropylene tube (Sarstedt AG & Co. KG, Nümbrecht, Germany). Extraction solvent (MeCN/H_2_O + 0.1% FA, 85/15, v/v, 2.75 mL) was added, tubes were vortexed thoroughly, and the mycotoxins were extracted at 300 rpm for 60 min on a rotary shaker followed by 15 min in an ultrasonic bath. Finally, sample extracts were vortexed again, filtrated through 0.2-µm nylon membrane filters (Agilent Technologies, Waldbronn, Germany) and provided for UHPLC-TQMS analysis. First, a qualitative screening for mycotoxins in the samples was performed. Afterwards, certain samples were diluted with H_2_O + 0.1% FA for expected mycotoxin concentrations to lay within the target working range, and quantification of mycotoxins was carried out. Each sample analysis was performed in duplicate using two 1-cm^2^ pieces from different parts of the sample material to partially compensate for inhomogeneous distribution of mycotoxins in the sample materials.

### UHPLC-TQMS conditions and echo calibration

Analysis of mycotoxins extracts was performed with an UHPLC-TQMS system according to Lindemann et al. (2022). Briefly, chromatographic separation was performed using MeCN + 0.1% FA and H_2_O + 0.1% FA on a reversed-phase column (Nucleodur C18 Gravity-SB, 75 × 2 mm, 1.8 µm, Macherey–Nagel GmbH & Co. KG, Düren, Germany) equipped with a 4 × 2 mm pre-column of the same material. An EVOQ Elite TQMS (Bruker Daltonics GmbH & Co. KG, Bremen, Germany) was applied for the following mass spectrometric detection and operated in scheduled multiple reaction monitoring (sMRM) mode. Determination of method performance characteristics was confined to specification of limits of detection and quantification (LODs/LOQs) based on signal to noise (S/N) ratios and linearity. Calibration levels were analogous to the ones described in the previously mentioned publication and prepared in neat solvent. The following mycotoxins were included in the method: Aflatoxins B_1_, B_2_, G_1_, and G_2_ (AFB_1/2_, AFG_1/2_); altenuene (ALT); alternariol monomethyl ether (AME); alternariol (AOH); beauvericin (BEA); citrinin (CIT); deoxynivalenol (DON); enniatins A, A_1_, B, and B_2_ (ENA, ENA_1_, ENB, ENB_1_); fumonisin B_1_ (FB_1_); gliotoxin (GTX); (2’*R*-)ochratoxin A ((2’*R*-)OTA); penitrems A and E (PEN A/E); sterigmatocystin (STG); T-2 toxin (T-2); HT-2 toxin (HT-2); zearalenone (ZEN); and the *Stachybotrys* toxins stachybotrychromenes A and B (STCHR A/B), satratoxins G and H (SAT G/H) and the PSDs stachybotrydial (STDIAL), stachybotrydial acetate (STDIAL AC), 2α-acetoxystachybotrydial acetate (ACDIAL AC), L-671,667 (L-671)s, stachybotrysin B (ST B), stachybotrysin C (ST C), stachybonoid D (STBON D), stachybotrylactam (STLAC), stachybotrylactam acetate (STLAC AC), and stachybotryamid (STAM) (see Table S1, Supplementary Information for chemical structures). Certain mycotoxin standards were isolated as part of previous projects and not commercially acquired. Specific information on purity can be found in the original publications listed in Lindemann et al. (2022). Calibration curves of each mycotoxin consisted of 4–8 calibration levels within the working range except for ENB and ENB_1_, for which only three levels were considered. The data can be found in the Supplementary Information (Table S2).

Assessment of emerging matrix effects during mass spectrometric detection was carried out in samples containing quantifiable mycotoxin contents applying the echo-peak technique established by Zrostlíková et al. (2002). The approach is based on a second injection of an analyte standard solution within the UHPLC-TQMS run of the sample. To enable a second injection, a different separation and injection system was coupled to the TQMS. For echo analysis chromatographic separation was performed using an Agilent 1100/1200 LC system (Agilent). Furthermore, an additional 4 × 2-mm pre-column (Nucleodur C18 Gravity, 3.0 µm, Macherey–Nagel) was installed at the MS valve. The technical setup applied during echo-peak analyses is illustrated in Fig. S1 in the Supplementary Information.

During echo-peak analysis, the applied MS method was reduced to the 14 mycotoxins, which were quantitated applying the previously described methodology. The adapted parameters are presented in Table S3 of the Supplementary Information. The detailed procedure of the echo approach was performed as follows: The injection of the sample solutions (not diluted, 30 µL) was set as the starting point of each analysis (*t* = 0.0 min). In the first 5 min of each run, the LC eluent was transferred directly to the UHPLC column and the TQMS system. Afterwards, the position of the valve at the TQMS switched, and the flow was additionally pumped through the pre-column prior to the UHPLC column. During an elaborated injection program, a neat solution containing a defined mycotoxin concentration was injected after a certain wait time for creating the echo peaks. Depending on the retention time of the mycotoxins, varying wait times needed to be applied to ensure an appropriate chromatographic separation of the analyte and corresponding echo peak (see Table S3, Supplementary Information). For most analytes, 30 µL of a medium calibration level were injected at a wait time of 6.5 min. Signal suppression and enhancement [SSE (%)] of different building materials on the echo peaks was determined by comparison to signal intensities of analogue mycotoxin concentrations in neat solutions. Effects on the analyte signal were compensated using the echo peak as an internal reference signal. A correction of determined mycotoxin concentrations in samples was performed only if the signal of the echo peak was altered by more than 30% in comparison to measurements in neat solution.

## Results and discussion

### Molds and CFUs in analyzed samples

**Fig. 1:**
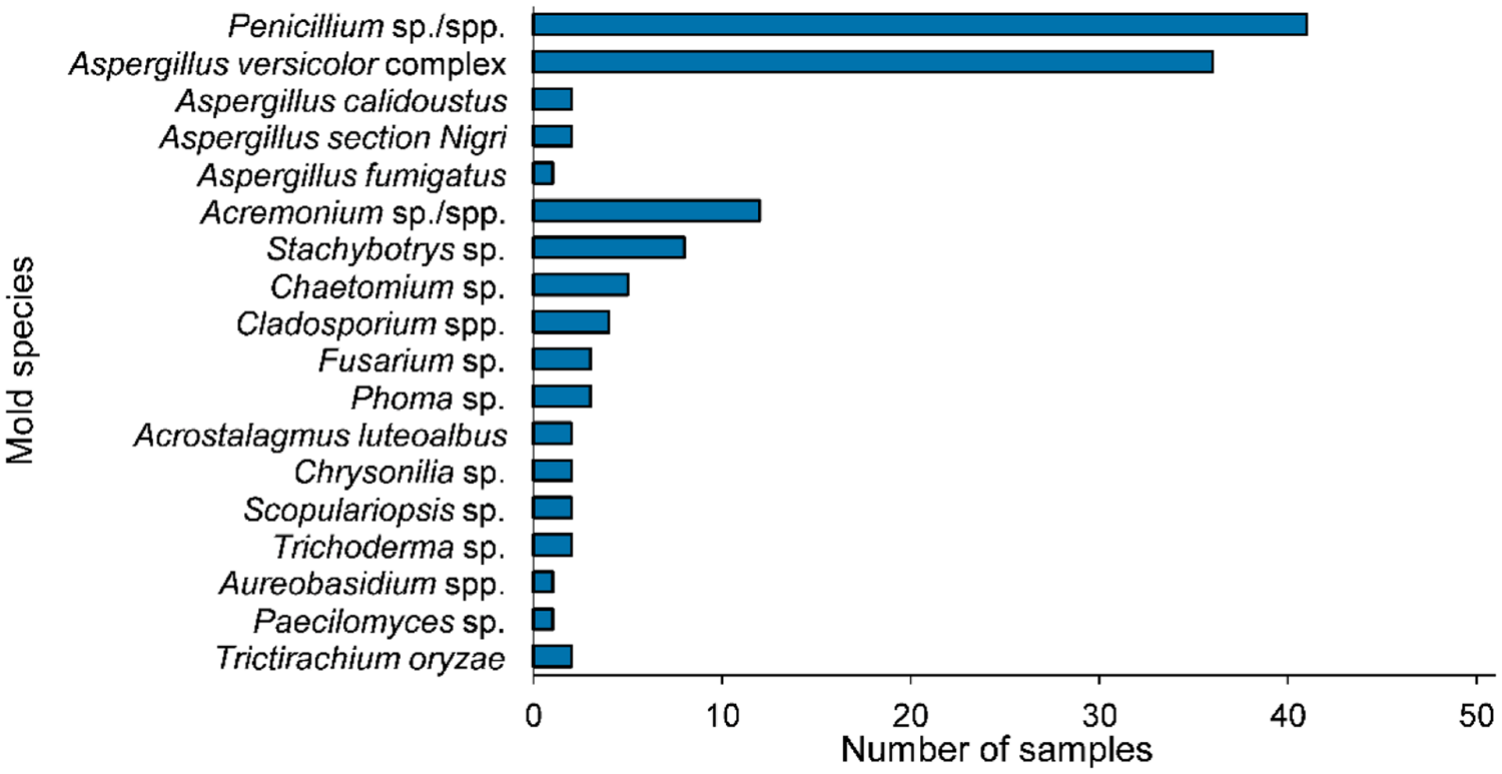
Distribution of identified mold genera/species in the investigated set of indoor building materials. Mold analysis was performed according to DIN ISO 16000–17:^2010^–06 standards. Corresponding data are presented in Table 1 of the Supplementary Information

Microbiological analysis of the 51 naturally mold infested indoor building samples revealed the presence of a large variety of different mold genera and species. Overall, 18 separate species were identified belonging to 15 independent genera. The distribution of the different molds in the samples is presented graphically in Fig. 1. In most samples, *Penicillium* sp./spp. (sp.: species, spp.: species pluralis) (*n* = 41) and *Aspergillus versicolor* complex (*n* = 36) were present. Among the *Aspergillus* species, three others were detectable in two (*Aspergillus calidoustus* and *Aspergillus section Nigri*) and one sample (*Aspergillus fumigatus*), respectively. Other typical indoor molds were also observed in the investigated samples including *Acremonium* sp./spp. (*n* = 12), *Stachybotrys* sp. (*n* = 8), *Chaetomium* sp. (*n* = 5), and *Cladosporium* spp. (*n* = 4). Furthermore, the following molds were identified in a decreasing number of samples: *Fusarium* sp. and *Phoma* sp. were present in three samples. *Acrostalagmus luteoalbus*, *Chrysonilia* sp., *Scopulariopsis* sp., and *Trichoderma* sp. in two samples and *Aureobasidium* spp., *Paecilomyces* sp., and *Tritirachium oryzae* were detectable in one sample. Overall, the determined prevalence and distribution of mold genera in the infested samples complies well with literature data (Andersen et al. 2011; Hyvärinen et al. 2002; Reboux et al. 2009). One noteworthy observation was that, in contrast to previous studies, *Stachybotrys* species were also detected on styrofoam samples and not only on cellulose-rich materials like wallpaper. No culturable fungi were traceable in seven samples. Table 1 contains detailed information about the molds present in the sample set.

In order to compare the extent of mold contamination in the samples, CFUs were determined according to DIN ISO 16000–17:^2010^–06 regulations. The results summarized in Table 1 indicate that the mold contamination varied greatly between the samples as viable fungi were detectable in a broad range from 10^2^ to 10^6^ CFU/g. These findings are in a common range for the conducted determination (Hyvärinen et al. 2002). Furthermore, differences in contamination with different mold genera occurred within certain samples: In sample no. 2, for example, a comparably low amount of *Penicillium* spp. (6.0 × 10^3^ CFU/g) paired with a high level of *Stachybotrys* sp. contamination (8.0 × 10^6^ CFU/g) was determined (compare Fig. 1).

### Analysis of mycotoxins in building materials

**Fig. 2:**
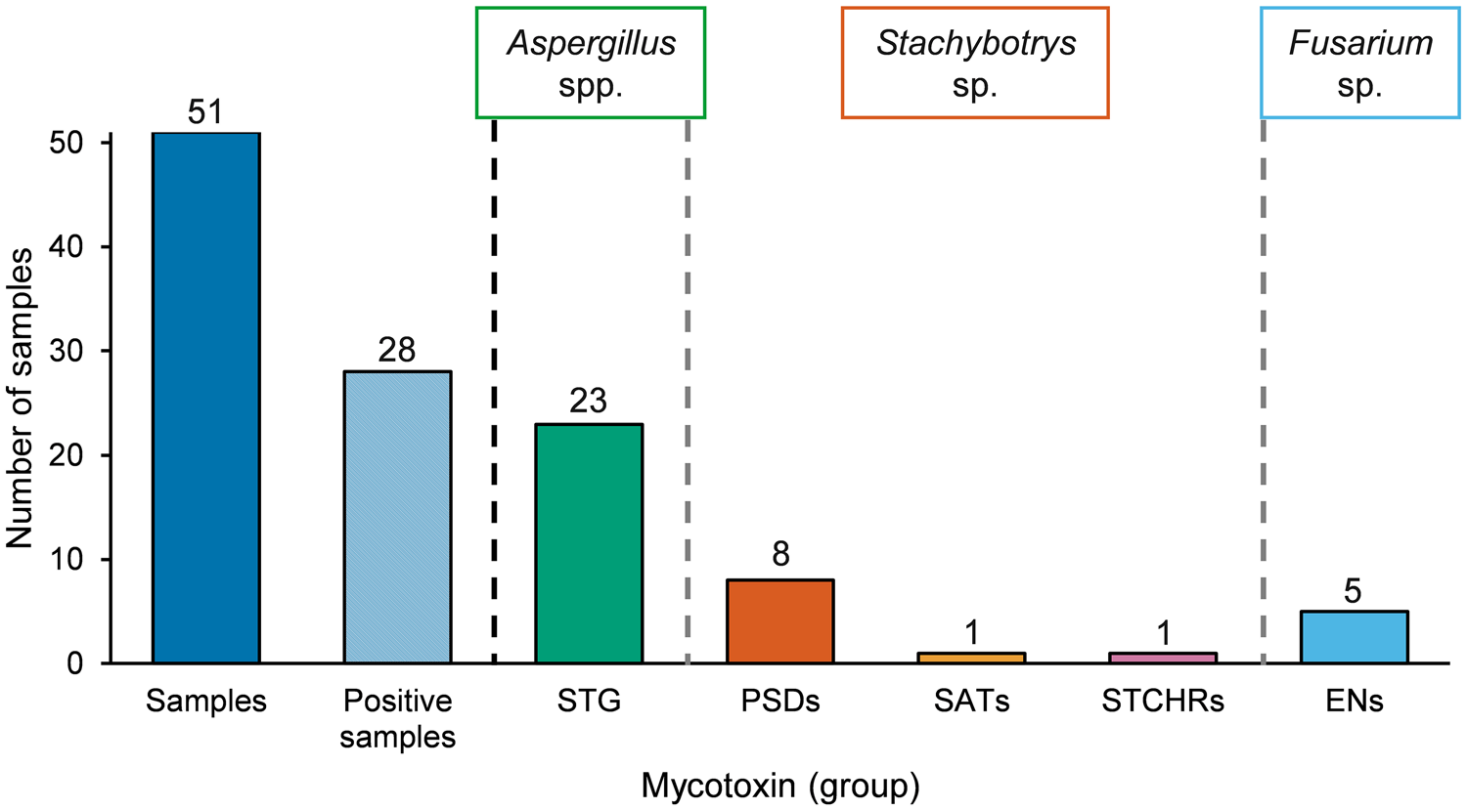
Investigated indoor material samples, in which mycotoxins and classes of mycotoxins derived from the mold genera *Aspergillus*, *Stachybotrys*, and *Fusarium* have been detected. Corresponding data are presented in Table 2

The analysis of mycotoxins in the sample set was carried out using a previously published instrumental UHPLC-TQMS approach (Lindemann et al. 2022). Further details are presented in Table S2 of the Supplementary Information. Calibration and quantification were carried out in neat solvent solutions as the sample set of interest consisted of a diverse spectrum of building materials, which made matrix-matched calibration hardly applicable. Preliminary analyses confirmed this assumption by additionally revealing largely varying compositions and contents of mycotoxins in different samples. The spectrum of considered mycotoxins in TQMS analysis covered 38 secondary metabolites from the most relevant mold species detected during microbiological analyses, including *Penicillium* spp., *Aspergillus* spp., *Stachybotrys* spp., and *Fusarium* spp. (see Table S1 for analyzed mycotoxins including chemical structure). These mycotoxins were determined in 28 of the naturally mold infested samples (compare Table 1), while no contamination was observed in 23 samples. Overall, 16 different mycotoxins including STG, ten different PSDs, two satratoxins, one stachybotrychromene, and two enniatins were detectable. A maximum of 13 mycotoxins was detected in one sample (sample no. 2) and 5 samples contained more than 10 mycotoxins (Table 1). The number of positive samples for each mycotoxin (group) is summarized in Fig. 2.

STG was the most prevalent mycotoxin in the investigated set of indoor building materials as it was detectable in 45% of all samples. In this case, results of microbiological and chemical analysis fit well as *Aspergillus versicolor* complex, which was also present in a majority of samples, is the most prominent producer of STG (EFSA 2013). However, it needs to be mentioned that the samples, in which STG was found and those in which *Aspergillus* species were identified, do not match completely. Four samples were positive for the aflatoxin-precursor without the proven presence of a corresponding mold species.

Previous studies concerning *Stachybotrys* toxins in indoor environments mostly focused on MCTs such as satratoxins as they are known to be extremely toxic to animals and humans (Eppley and Bailey 1973; Nielsen et al. 2009). However, only a fraction of all *Stachybotrys* strains is able to produce these compounds, while other metabolites, namely the PSDs, are expected to be formed by all strains (Andersen et al. 2002; Ulrich et al. 2020). This described distribution of mycotoxins derived from *Stachybotrys* species was verified in the presented study. In TQMS analysis, PSDs were traceable in eight of the building material samples (about 16%). All eight samples contained at least one PSD, and in five samples, the full variety of the ten investigated PSDs was present. In contrast to this, SAT G and H were only identified on one wallpaper. In this sample, STCHR B was present, as well. Again, these results are in good accordance with the data obtained from the culturable mold determination experiments. In eight samples, *Stachybotrys* sp. were identified during microbiological analyses. Two of these samples did not show traces of PSDs during mycotoxin determination; however, two additional samples were tested positive for *Stachybotrys* toxins without *Stachybotrys* being identified beforehand.

The last group of secondary metabolites observed in the sample set was derived from the genus *Fusarium*. Two enniatins occurred in different building materials: ENB was detectable in five samples, whereas ENA_1_ was present in one sample. The microbiological analyses verified traces of *Fusarium* species in three independent samples. No considered mycotoxins of *Penicillium* species like citrinin, ochratoxin A, or penitrems were identified in the presented study, even though most samples were positive for microfungi of this genus. However, this is not an unusual observation. Tuomi et al. also noticed a discrepancy between samples showing *Penicillium* infestation and those that actually contained *Penicillium* toxins (Tuomi et al. 2000). The applied agar during routine determination of culturable mold can be a critical point as some media provide favorable conditions for certain mold genera. Ultimately, the choice of MEA can have an impact on the prevalence of *Penicillium* and *Aspergillus* compared to other genera as this medium is preferred by these fast-growing molds (Andersen and Nissen 2000). One further possible explanation is that *Penicillium* toxins were not produced under the existing conditions in the investigated samples or produced in rather low quantities. Additionally, it is possible that the samples contained mycotoxins, which were not covered by the applied UHPLC-TQMS method.

Overall, the determination of mold genera and the associated mycotoxins matched in 24 of 51 samples. As previously discussed, the detection of mycotoxins without the verified presence of the respective mold occurred in ten of the monitored samples. Reasons for this observation are manifold: A low amount of biomass of the fungus could have led to a non-detect in microbiological analyses, for example, or the mycotoxins could have been introduced to the sample through other paths. An elevated sensitivity of TQMS detection compared to microbiological analysis is also plausible considering the results of enniatin and *Fusarium* determination. Also, an adaption of the applied standard protocol used for CFU determination (DIN ISO 16000–17:^2010^–06) might be reasonable so that a uniform sample-weight is considered rather than a uniform area especially for samples with low densities such as styrofoam. A discrepancy manifested by the fact that, for example, molds such as *Stachybotrys*, for which MEA is not an ideal culture medium, are discriminated during fungal cultivation, but in contrast, a high number of positive samples for *Stachybotrys* toxins is detected, was not observed. However, the determination of a mold genera or species without the detection of (certain) mycotoxins in the samples was much more common than vice versa. As mentioned for the genus *Penicillium*, no or rather low mycotoxin contents below the limits of detection of the TQMS approach could be accountable for this observation. In the future, this problem could be overcome by extracting larger quantities of infested materials. This would also have positive effects on the homogeneity and representativeness of the results. However, as the linkage between mycotoxin contamination on surfaces and mycotoxin exposure is by far more complex, the relevance of small shifts of mycotoxin contamination patterns appears neglectable. Regarding the variety of verified microfungi in the investigated sample set and considering the resulting number of potentially formed mycotoxins, it is more likely that further present analytes like roridin A or other PSDs were simply not covered by the applied UHPLC-TQMS method.

### Quantification of mycotoxins and observed matrix effects

**Fig. 3:**
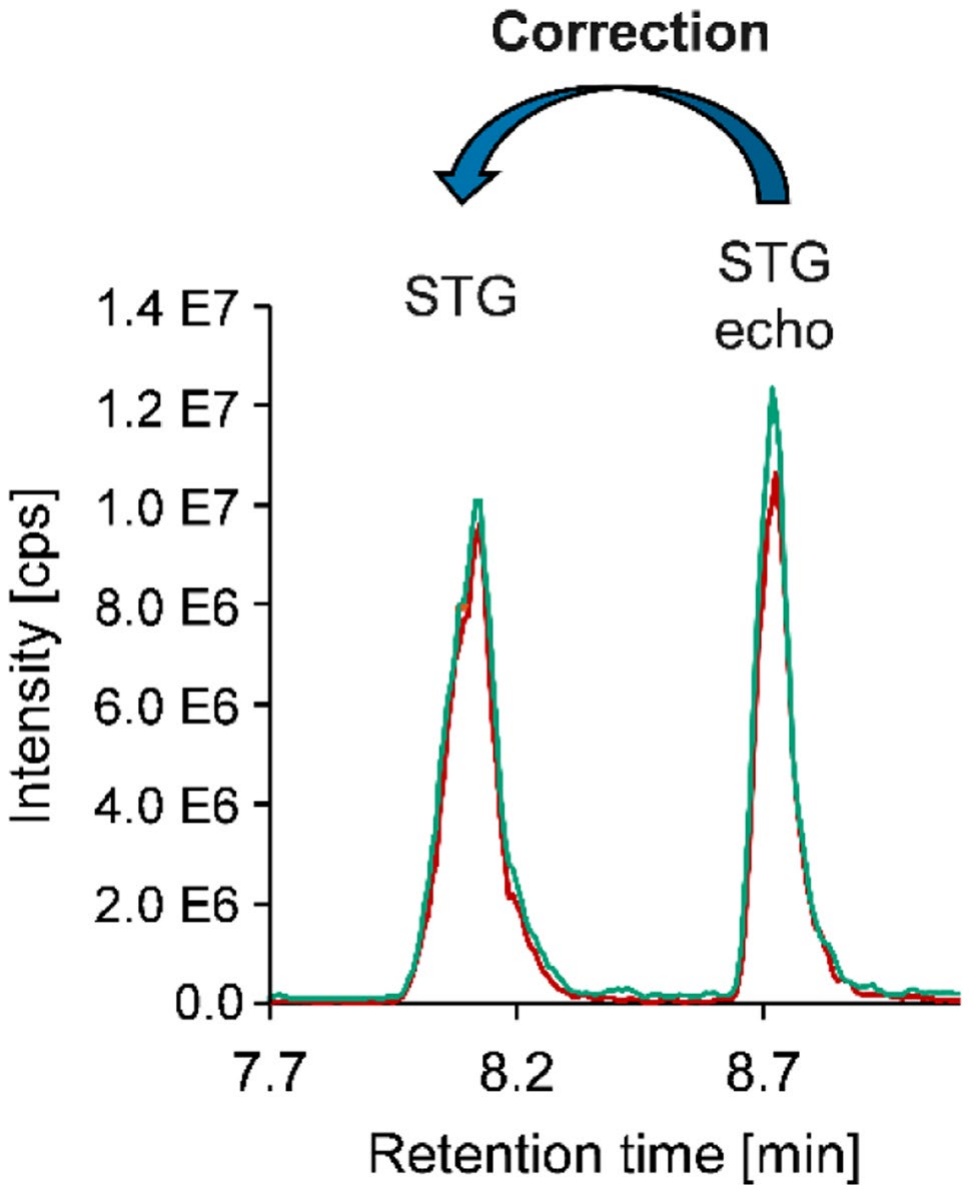
Principle of the echo-peak technique. Analyte (STG) and echo peak (STG echo) of the mycotoxin STG in the UHPLC-TQMS run of a naturally contaminated sample are chromatographically separated but elute in close proximity. Matrix effects on the echo peak are evaluated by comparison with measurements in neat solutions and enable a correction of the analyte peak (green, quantifier transition (325.1 → 281.0); red, qualifier transition (325.1 → 310.0))

Vishwanath et al. (2009) analyzed the effects of matrix components of mortar and carton-gypsum board on the mass spectrometric detection of mycotoxins and described them as negligible. Nevertheless, due to the heterogeneity of the sample materials, matrix effects should not be completely ignored in the context of the presented study. Therefore, echo-peak calibration, a straightforward internal calibration approach, was performed to estimate potentially occurring effects on ionization and mass spectrometric detection. In particular, influences of different pre-treatments (color, paste, etc.) and of varying degrees of mold infestation of the building materials should be considered, as they have not been discussed thoroughly in literature yet. For this purpose, the 25 samples containing quantifiable mycotoxins were re-analyzed applying a second injection of a mycotoxin standard solution to the UHPLC-TQMS run according to the echo-peak technique described in detail in the “Materials and methods” section and in Fig. S1 and Table S3 in the Supplementary Information (Zrostlíková et al. 2002). By additionally injecting the corresponding standard, a second peak of the mycotoxin of interest, the so-called echo peak, is created. The echo peak is chromatographically separated from but in close proximity to the peak deriving from the naturally contaminated sample, as shown exemplary for the mycotoxin STG in Fig. 3. Matrix effects of sample components on the echo peak are determined by comparison to measurements in neat solvent. The assumption is that both the echo peak and the analyte peak are affected by comparable matrix effects as they elute in close proximity. Therefore, a calculation of signal suppression and enhancement [SSE (%)] of the analyte peak in the sample respectively a correction of the analyte results can be performed using the echo peak as an internal reference. The threshold value for the correction was a deviation of the area of the echo signal by more than 30% compared to measurements in solvent.

Overall, moderate SSE values (< 30%) of the mycotoxin echo signals were determined. An alteration of signals by more than 30% was observable in seven samples, affecting 15 mycotoxin signals altogether. In 13 cases, the alteration was classified as a suppression and for two mycotoxin matrix components lead to enhanced signals of the echo peak. The strongest signal suppression was observed for ACDIAL AC in sample no. 23 (23.4%), and the highest signal enhancement was calculated for STG in sample no. 49 (156.5%). There were certain samples with rather complex matrices, as a correction of all quantifiable mycotoxins was necessary according to the results of echo peak analyses (e.g., sample no. 21). However, there were also samples, like sample no. 2, in which stronger matrix interferences were limited to some mycotoxins. Generally, PSDs containing a (reactive) dialdehyde moiety (ACDIAL AC, STDIAL AC, STDIAL) showed a tendency for signal suppression. Complete results on determined SSE values are summarized in Table S4 of the Supplementary Information. As mentioned above, quantification of mycotoxins in the investigated building material samples was based on calibration using neat mycotoxin solutions. The determined—and partially echo peak corrected—mycotoxin levels in the analyzed set of naturally mold-infested building materials are summarized in Table 2 (for details see Table S5 in the Supplementary Information).

**Table 2:** Mycotoxin contents in analyzed indoor building material samples determined by UHPLC-TQMS (*n* = 51). If necessary, echo peak correction was performed (compare Table S4). Respective mycotoxin abbreviations are listed in Table S1

Mycotoxin	Positive samples [%] (*n*)	Quantified samples [%] (*n*)	Content [ng/cm^2^]		
			**Mean **^**a**^	**Median **^**a**^	**Maximum**
**ACDIAL AC**^**b**^	11.8 (6)	11.8 (6)	1599	445	7740
**ENA**_**1**_	2.0 (1)	-			
**ENB**	9.8 (5)	5.9 (3)	0.352	0.365	0.369
**L-671**^**b**^	13.7 (7)	13.7 (7)	49	25	161
**SAT G**	2.0 (1)	2.0 (1)	1381^c^		
**SAT H**	2.0 (1)	2.0 (1)	1267^c^		
**STBON D**^**b**^	15.7 (8)	13.7 (7)	128	21	691
**ST B**	9.8 (5)	9.8 (5)	69	39	153
**ST C**	11.8 (6)	11.8 (6)	26.4	17.9	76.9
**STAM**	9.8 (5)	5.9 (3)	60	10	164
**STCHR B**^**b**^	2.0 (1)	-			
**STDIAL**^**b**^	11.8 (6)	11.8 (6)	302	66	1468
**STDIAL AC**^**b**^	9.8 (5)	9.8 (5)	660	313	2377
**STG**^**b**^	45.1 (23)	41.2 (21)	48	1	979
**STLAC**^**b**^	9.8 (5)	7.8 (4)	1329	586	3863
**STLAC AC**	9.8 (5)	7.8 (4)	92	62	218

^a^Mean and median were calculated from samples > LOQ

^b^Echo correction was performed for certain samples

^c^Only one quantifiable sample

Among the group of cytotoxic enniatins derived from *Fusarium* species (Firáková et al. 2007), only ENB was present in three samples (samples no. 10, 12, 24) at levels of 0.323–0.369 ng/cm^2^. The concentration of ENA_1_ was below the limit of quantification and therefore not quantified. Neither quantifiable nor qualitative results of *Fusarium* toxin analysis were related to the culturable mold experiments, which emphasizes the fact that the presence of mold is not necessarily equivalent to the occurrence of mycotoxins.

The potentially carcinogenic *Aspergillus* toxin STG (IARC 1976, 1987) was quantifiable in 21 of 23 positive samples. Overall, STG contents covered a broad range (0.516–979 ng/cm^2^) with the median at 1 ng/cm^2^. Samples with elevated STG contamination showed values between about 3 ng/cm^2^ (samples no. 15, 29, 49) and 10 ng/cm^2^ (sample no. 30). Determined CFU counts of *Aspergillus versicolor* complex in the samples varied between 10^3^ and 10^4^ per gram. The highest STG contamination was calculated for sample no. 9 with 979 ng/cm^2^. Here, an elevated count of culturable *Aspergillus versicolor* complex of 7.0 × 10^5^ CFU/g was determined. However, even higher exposures in the 10^6^ CFU/g range were identified in further samples (sample nos. 5, 6, 7), which at the same time showed lower levels of STG (about 0.9–2.2 ng/cm^2^). Therefore, again, a correlation between determined amounts of *Aspergillus* toxins and fungal exposure was not observed.

Concerning mycotoxins derived from *Stachybotrys*, analyte contents of two of the three investigated compound classes (MCTs, PSDs) were calculated. The levels of the two satratoxins SAT G and SAT H were determined in one wallpaper sample (sample no.2). For both compounds, comparable values of about 1.3 µg/cm^2^ were determined.

Of the eight samples containing PSDs, seven showed quantifiable STBON D and L-671 levels. ACDIAL AC, ST C, and STDIAL were quantified in six building materials, whereas ST B and STDIAL AC were present in five samples at levels > LOQ. Additionally, the extracts of four building materials contained STLAC and STLAC AC in quantifiable concentrations. STAM was quantified in three samples. Overall, the investigated samples showed higher contents of PSDs compared to STG and ENB. Lowest mean PSD amounts were calculated for the mycotoxin ST C with 26.4 ng/cm^2^. The maximum contamination of *Stachybotrys* toxins was determined in sample no. 2, in which, besides the already discussed satratoxins and STCHR B, all investigated PSDs were present at high levels. Four PSDs were present above 1 µg/cm^2^ with the maximum determined for ACDIAL AC at about 7.7 µg/cm^2^ (compare Table 2 and Table S5, Supplementary Information). Compared to the other samples containing *Stachybotrys* toxins (styrofoam), sample no. 2 is a cellulose-rich matrix (wallpaper), which seems to stimulate the fungal mycotoxin production. In summary, the determined levels were slightly lower compared to those reported by Jagels et al. but still within a comparable range (Jagels et al. 2020). However, especially for PSDs, an inhomogeneity of determined mycotoxin levels in duplicate determinations was observed for some samples, resulting in increased standard deviations (compare Table S5) and indicating a not completely representative sampling.

Besides the highest amounts of *Stachybotrys* toxins, sample no. 2 additionally showed the highest CFU counts of *Stachybotrys* (8.0 × 10^6^ CFU/g). Contrary to the mold genera discussed above, a positive relationship between *Stachybotrys* toxins and CFUs of the respective mold in the sample set was observed. Samples indicating a higher *Stachybotrys* infestation due to elevated CFU counts in a range of 10^5^–10^6^ CFU/g (samples no. 2, 5, 6, 7, 8) contained more PSDs and higher levels compared to samples with lower infestation (sample no. 21: 2.0 × 10^4^ CFU/g; five PSDs identifiable). In building materials with calculated CFU values below 10^4^ CFU/g of *Stachybotrys* (samples no. 13, 14), no *Stachybotrys* toxins were detected. These observations may potentially be related to the fact that the number of mycotoxins considered for *Stachybotrys* species was significantly higher compared to any other mold investigated.

The presented study was conducted to characterize a set of naturally mold infested indoor building materials regarding traceable mold and mycotoxin levels. In agreement with previous publications, a large number of diverse (indoor) molds was identified showing broad CFU ranges (Andersen et al. 2011; Hyvärinen et al. 2002; Reboux et al. 2009). Fungi of the genera *Penicillium* and *Aspergillus* were particularly present, which was also reflected by the corresponding mycotoxin determination by UHPLC-TQMS. Overall, 45% of all samples were tested positive for the *Aspergillus* toxin STG. These data suggest that there is an increased occurrence of this potentially carcinogenic mycotoxin indoors and that it should therefore be of particular concern. Further mycotoxins detected within the study mainly originated from the toxic indoor mold *Stachybotrys*. Concerning this genus, hypotheses of recent studies (Jagels et al. 2020) were confirmed as PSDs were detected frequently and at high levels, whereas MCTs played a subordinate role and were detected only in one sample.

The applied UHPLC-TQMS approach is suitable for the purpose of this study as the sensitive detection of mycotoxins in the diverse sample set was enabled. The applied sampling method of two 1-cm^2^ subsamples can be regarded as insufficient for a complete description of the mycotoxin pattern of the whole contaminated building material. Nevertheless, the general mycotoxin profile could be clearly assigned. Overall, low matrix effects of building materials on mass spectrometric detection were observed. Therefore, solvent calibration in combination with echo-peak correction was an applicable approach for mycotoxin quantification. However, future studies targeting a sole screening of mycotoxins in non-complex sample materials may even renounce this additional correction step. The infested samples did not show an unusual high level of decomposition nor were they taken from residences, uninhabitable due to mold infestation. It can therefore be assumed, that the determined CFU counts, and mycotoxin contents in the analyzed building materials represent a realistic scenario of their indoor occurrence. A wide range of mycotoxins was detected in the analyzed sample set, and an exceptionally high prevalence of PSDs derived from *Stachybotrys* species was demonstrated. To evaluate toxic effects of the identified mycotoxins, different ways of human uptake have to be considered as they can tremendously influence toxicity (Creasia et al. 1990; Jakšić et al. 2020). For several mycotoxins, like PSDs, potentially occurring health effects have not even been sufficiently characterized in vitro or in vivo yet (Ma et al. 2018).

The question that inevitably arises whenever data on indoor mold presence and mycotoxin contamination are introduced is to what extent they have an impact on human health. The presented data emphasized that a presence of mold is not necessarily equivalent to the occurrence of mycotoxins. Additionally, the determination of indoor mold and mycotoxin occurrence on building materials does not enable any direct conclusions on human exposure or potential health risks. However, it is a first step in characterizing those mold species and secondary metabolites, which may be of relevance indoors. Emerging health effects of residents of mold-infested housing can only be clarified if mycotoxin research in residential settings is as consequently performed as mycotoxin exposure studies via the food chain have been over the past decades. Attempts should be made to correlate indoor mycotoxin exposures with adverse health impairments of occupants of infested buildings. Additionally, houses without mold infestation should be included as controls in future studies. For this purpose, human indoor exposure biomarkers, as used extensively for mycotoxin exposure by food intake, might be helpful (Schmidt et al. 2021a, 2021b). The present study clearly indicates that there are still several additional questions to be addressed in this context. For instance, comparatively few mycotoxins were identified in the investigated samples despite a large variety of molds being detected, and in 19 samples, none of the analyzed mycotoxins were detectable despite verified mold contamination. In future projects, the occurrence of further mycotoxins in the interior environment should therefore be examined by increasing the number of considered analytes. The combination of chromatographic separation on a reversed-phase UHPLC-column and TQMS detection applied in the presented study is suitable for this purpose. Furthermore, detection using high-resolution mass spectrometric devices and creating data sets, which can additionally be analyzed retrospectively for new, emerging mycotoxins represents an important amendment of the analytical approach. Besides the analytical challenges, there are numerous toxicological aspects to be resolved before a final human exposure and hazard assessment of mycotoxins in indoor environments will be feasible.

## Supplementary information

Below is the link to the electronic supplementary material.

Supplementary file1 (DOCX 454 KB)

## Data Availability

All data are available in the manuscript and the Supplementary Information.

## Abbreviations

ACDIAL AC: 2α-Acetoxystachybotrydial acetate
CFU: Colony-forming unit
DG: Dichloran glycerin
DIN: German Institute for Standardization
EN: Enniatin
FA: Formic acid
ISO: International Organization for Standardization
L-671: L-671,667
MCTs: Macrocyclic trichothecenes
MEA: Malt extract agar
PSDs: Phenylspirodrimanes
STG: Sterigmatocystin
SAT: Satratoxin
STAM: Stachybotryamid
ST B: Stachybotrysin B
STBON D: Stachybonoid D
ST C: Stachybotrysin C
STCHR: Stachybotrychromene
STDIAL: Stachybotrydial
STDIAL AC: Stachybotrydial acetate
STLAC: Stachybotrylactam
STLAC AC: Stachybotrylactam acetate
SSE: Signal suppression and enhancement
TQMS: Triple-quadrupole mass spectrometer
UHPLC: Ultra-high performance liquid chromatography
