# Supplementary material for: Analysis of mold and mycotoxins in naturally infested indoor building materials
Source: Mycotoxin Res. 2022 Jul 28;38(3):205–20. doi: 10.1007/s12550-022-00461-3 (PMC9356937; doi:10.1007/s12550-022-00461-3)
Supplement: Supplementary file 1 — Supplementary file1 (DOCX 454 KB) [file 12550_2022_461_MOESM1_ESM.docx]

**Supplementary Information**

**Analysis of Mold and Mycotoxins in Naturally Infested Indoor Building Materials**

Viktoria Lindemann^a^, Tim Schleiner^b^, Ulrich Maier^b^, Hubert Fels^b^, Benedikt Cramer^a^, Hans-Ulrich Humpf^a*^

^a^Institute of Food Chemistry, Westfälische Wilhelms-Universität Münster, Corrensstr. 45, 48149 Münster, Germany

^b^Umweltlabor ACB GmbH Münster, Albrecht-Thaer-Straße 14, 48147 Münster, Germany

*Corresponding author e-mail: [humpf@wwu.de](\\\\nwz.wwu.de\\dfs\\home\\v\\v_lind05\\Desktop\\Dateien Promotion\\Paper\\Untersuchung Innenraummaterialien\\humpf@wwu.de)

ORCID: Benedikt Cramer, 0000-0001-7632-8676;

Hans-Ulrich Humpf, 0000-0003-3296-3058

**Table S1:** Names, abbreviations, and chemical structures of investigated mycotoxins.

| Analyte | Abbreviation | Chemical structure |
| --- | --- | --- |
| 2’*R* Ochratoxin A | 2’*R* OTA |  |
| Ochratoxin A | OTA |  |
| 2α-Acetoxystachybotrydial acetate | ACDIAL AC |  |
| Aflatoxin B_1_ | AFB_1_ |  |
| Aflatoxin B_2_ | AFB_2_ |  |
| Aflatoxin G_1_ | AFG_1_ |  |
| Aflatoxin G_2_ | AFG_2_ |  |
| Altenuene | ALT |  |
| Alternariol monomethyl ether | AME |  |
| Alternariol | AOH |  |
| Beauvericin | BEA |  |
| Citrinin | CIT |  |
| Deoxynivalenol | DON |  |
| Enniatin A | ENA |  |
| Enniatin A_1_ | ENA_1_ |  |
| Enniatin B | ENB |  |
| Enniatin B_1_ | ENB_1_ |  |
| Fumonisin B_1_ | FB_1_ |  |
| Gliotoxin | GTX |  |
| HT-2 toxin | HT-2 |  |
| L-671,667 | L-671 |  |
| Penitrem A | Pen A |  |
| Penitrem E | Pen E |  |
| Satratoxin G | SAT G |  |
| Satratoxin H | SAT H |  |
| Stachybotrysin B | ST B |  |
| Stachybotrysin C | ST C |  |
| Stachybotryamid | STAM |  |
| Stachybonoid D | STBON D |  |
| Stachybotrychromene A | STCHR A |  |
| Stachybotrychromene B | STCHR B |  |
| Stachybotrydial acetate | STDIAL AC |  |
| Stachybotrydial | STDIAL |  |
| Sterigmatocystin | STG |  |
| Stachybotrylactam | STLAC |  |
| Stachybotrylactam acetate | STLAC AC |  |
| T-2 toxin | T-2 |  |
| Zearalenone | ZEN |  |

**Table S2:** Method performance characteristics of the developed TQMS method for the detection of mycotoxins in neat solvent solutions. Respective mycotoxin abbreviations are listed in Table S1. (LOD: limit of detection, defined at a S/N ratio of 3; LOQ: limit of quantification, defined at a S/N ratio of 10.)

| Analyte | LOD [ng/mL] | LOQ [ng/mL] | Working range [ng/mL] | Weighting | R^2^ |
| --- | --- | --- | --- | --- | --- |
| 2’*R* OTA | 0.900 | 3.00 | 5.00–100 | 1/x | 0.994 |
| ACDIAL AC | 10.3 | 34.2 | 37.5–750 | 1/x | 0.995 |
| AFB_1_ | 0.042 | 0.140 | 0.250–50.0 | 1/x | 0.992 |
| AFB_2_ | 0.016 | 0.053 | 0.075–15.0 | 1/x | 0.996 |
| AFG_1_ | 0.028 | 0.093 | 0.250–50.0 | 1/x | 0.993 |
| AFG_2_ | 0.019 | 0.063 | 0.075–15.0 | 1/x | 0.998 |
| ALT | 0.038 | 0.127 | 2.50–500 | 1/x | 0.992 |
| AME | 0.179 | 0.597 | 1.25–50.0 | 1/x | 0.993 |
| AOH | 0.306 | 1.02 | 2.50–500 | 1/x | 0.995 |
| BEA | 0.150 | 0.500 | 0.630–2.50 | 1/x | 0.993 |
| CIT | 0.066 | 0.220 | 2.50–500 | 1/x | 0.994 |
| DON | 0.703 | 2.34 | 3.13–625 | 1/x | 0.994 |
| ENA | 0.966 | 3.22 | 4.00–40.0 | 1/x | 0.987 |
| ENA_1_ | 0.903 | 3.01 | 4.00–40.0 | 1/x | 0.991 |
| ENB | 0.023 | 0.077 | 0.125–0.500 | 1/x | 0.998 |
| ENB_1_ | 0.054 | 0.180 | 0.250–1.00 | 1/x | 0.994 |
| FB_1_ | 0.300 | 1.00 | 5.00–1000 | 1/x | 0.990 |
| GTX | 0.058 | 0.193 | 0.750–150 | 1/x | 0.993 |
| HT-2 | 0.330 | 1.10 | 3.00–600 | 1/x | 0.996 |
| L-671 | 1.06 | 3.53 | 6.25–250 | 1/x | 0.990 |
| OTA | 0.900 | 3.00 | 5.00–100 | 1/x | 0.994 |
| PEN A | 0.990 | 3.30 | 5.00–50.0 | 1/x | 0.998 |
| PEN E | 2.76 | 9.21 | 12.5–250 | 1/x | 0.990 |
| SAT G | 0.130 | 0.433 | 0.750–150 | 1/x | 0.991 |
| SAT H | 0.140 | 0.467 | 1.50–300 | 1/x | 0.993 |
| ST B | 1.70 | 5.65 | 7.50–150 | 1/x | 0.990 |
| ST C | 1.01 | 3.36 | 3.75–150 | 1/x | 0.994 |
| STAM | 0.750 | 2.50 | 2.50–50.0 | 1/x | 0.994 |
| STBON D | 0.726 | 2.42 | 2.50–50.0 | 1/x | 0.989 |
| STCHR A | 24.9 | 83.1 | 100–10000 | - | 0.989 |
| STCHR B | 11.0 | 36.6 | 62.5–2500 | 1/x | 0.999 |
| STDIAL AC | 5.62 | 18.7 | 37.5–150 | 1/x | 0.993 |
| STDIAL | 2.72 | 9.07 | 12.5–500 | 1/x | 0.994 |
| STG | 0.057 | 0.190 | 0.250–5.00 | 1/x | 0.994 |
| STLAC | 20.6 | 68.7 | 125–2500 | 1/x | 0.992 |
| STLAC AC | 2.91 | 9.70 | 10.0–100 | 1/x | 0.994 |
| Table S2: continued from previous page. | | | | | |
| Analyte | **LOD [ng/mL]** | **LOQ [ng/mL]** | **Working range [ng/mL]** | **Weighting** | **R^2^** |
| T-2 | 0.919 | 3.06 | 5.00–1000 | 1/x | 0.993 |
| ZEN | 4.27 | 14.2 | 31.3–1250 | 1/x | 0.991 |


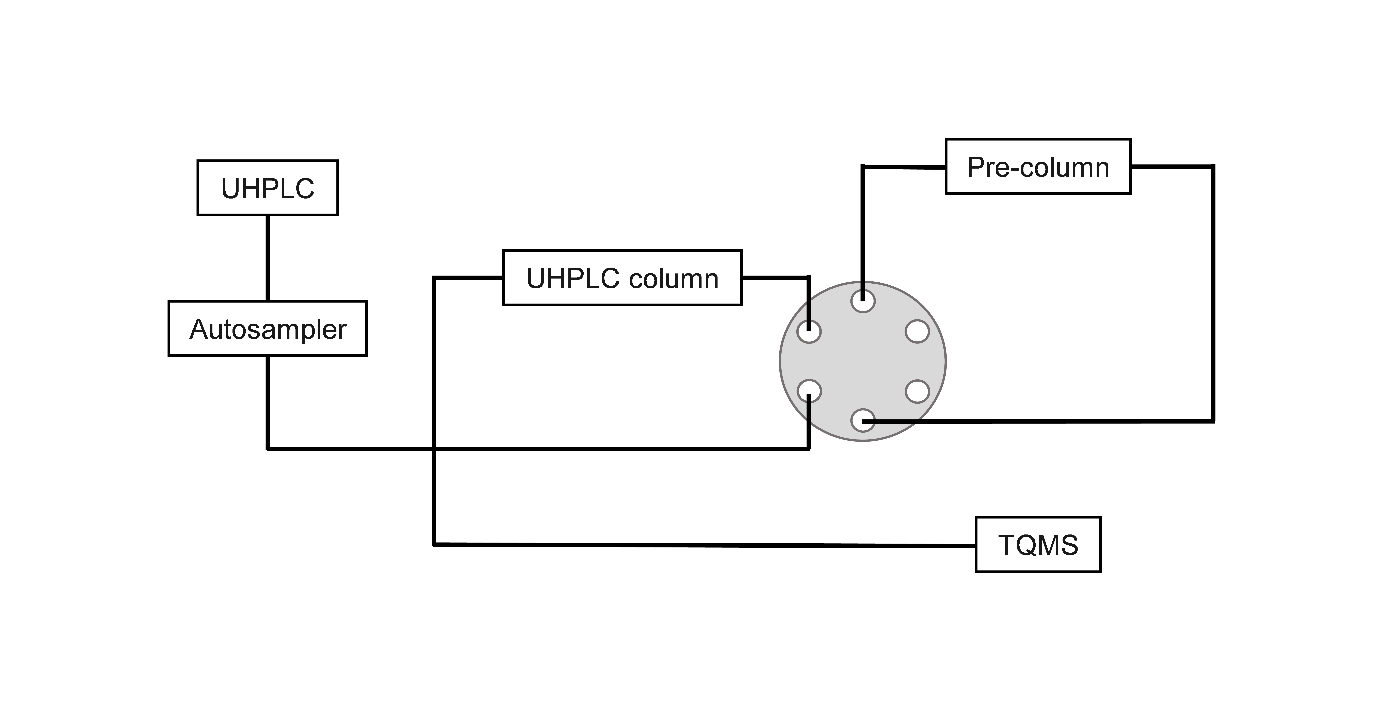


**Figure S1:** Instrumental setup for measurements applying the echo-peak technique. An additional pre-column is added to the system to prevent coelution of analyte and corresponding echo peaks (for further details see Material and Method section of the manuscript).

**Table S3:** Scheduled multiple reaction monitoring (sMRM) transitions, resolution of quadrupoles (Q1/Q3), collision energies (CE) and retention times (RT) of the TQMS method applied for echo peak analysis. The quantifier transition of each mycotoxin is highlighted. Respective mycotoxin abbreviations are listed in Table S1.

| Analyte | Q1 mass  [*m*/*z*] | Q1 resolution | Q3 mass  [*m*/*z*] | Q3 resolution | CE [eV] | Wait [min] | RT analyte peak [min] | RT echo peak [min] | RT set [min] |
| --- | --- | --- | --- | --- | --- | --- | --- | --- | --- |
| ACDIAL AC | 485.2 [M-H]^-^ | 1.0 | **399.0** | 2.5 | 23 | 6.5 | 8.9 | 9.4 | 9.2±2.0 |
|  |  | 1.0 | 357.0 | 2.5 | 27 |  |  |  | 9.2±2.0 |
|  |  | 1.0 | 365.0 | 2.5 | 32 |  |  |  | 9.2±2.0 |
|  |  | 1.0 | 150.0 | 2.5 | 45 |  |  |  | 9.2±2.0 |
| ENB | 640.4 [M+H]^+^ | 1.5 | **196.1** | 5.0 | -22 | 8.0 | 10.0 | 10.6 | 10.3±2.0 |
|  |  | 1.5 | 214.1 | 5.0 | -24 |  |  |  | 10.3±2.0 |
| L-671 | 389.2 [M+H]^+^ | 2.0 | **163.0** | 2.0 | -26 | 6.5 | 7.6 | 8.6 | 8.5±2.0 |
|  |  | 2.0 | 181.2 | 2.0 | -15 |  |  |  | 8.5±2.0 |
| SAT G | 545.2 [M+H]^+^ | 1.5 | **249.1** | 2.5 | -11 | 5.5 | 6.6 | 7.2 | 7.4±3.0 |
|  |  | 1.5 | 231.1 | 2.5 | -12 |  |  |  | 7.4±3.0 |
| SAT H | 529.2 [M+H]^+^ | 2.0 | **231.1** | 2.0 | -9 | 5.5 | 6.8 | 7.2 | 7.5±3.0 |
|  |  | 2.0 | 245.0 | 2.0 | -10 |  |  |  | 7.5 |
| ST B | 413.3 [M-H_2_O+H]^+^ | 2.0 | **163.0** | 2.0 | -24 | 6.5 | 8.5 | 9.1 | 9.1±2.0 |
|  |  | 2.0 | 145.1 | 2.0 | -38 |  |  |  | 9.1±2.0 |
| ST C | 429.2 [M-H_2_O+H]^+^ | 1.5 | **163.0** | 5.0 | -24 | 6.5 | 7.6 | 8.5 | 8.0±3.0 |
|  |  | 1.5 | 187.1 | 5.0 | -20 |  |  |  | 8.0±3.0 |
| STAM | 430.3 [M+H]^+^ | 1.5 | **222.1** | 2.5 | -27 | 6.5 | 7.5 | 8.4 | 7.8±3.0 |
|  |  | 1.5 | 260.0 | 2.5 | -29 |  |  |  | 7.8±3.0 |
| STBON D | 471.2 [M-H_2_O+H]^+^ | 2.0 | **163.0** | 2.0 | -25 | 6.5 | 8.0 | 8.7 | 8.5±3.0 |
|  |  | 2.0 | 187.1 | 2.0 | -26 |  |  |  | 8.5±3.0 |
| STDIAL AC | 427.2 [M-H]^-^ | 1.5 | **367.0** | 5.0 | 28 | 6.5 | 9.1 | 9.5 | 9.3±2.0 |
|  |  | 1.5 | 147.1 | 5.0 | 28 |  |  |  | 9.3±2.0 |
|  |  | 1.5 | 175.0 | 5.0 | 18 |  |  |  | 9.3±2.0 |
|  |  | 1.5 | 150.0 | 5.0 | 40 |  |  |  | 9.3±2.0 |
| STDIAL | 385.2 [M-H]^-^ | 1.5 | **122.0** | 2.5 | 10 | 6.5 | 8.3 | 8.8 | 8.8±2.0 |
|  |  | 1.5 | 341.2 | 2.5 | 19 |  |  |  | 8.8±2.0 |
|  |  | 1.5 | 313.2 | 2.5 | 25 |  |  |  | 8.8±2.0 |
|  |  | 1.5 | 150.0 | 2.5 | 35 |  |  |  | 8.8±2.0 |
| STG | 325.1 [M+H]^+^ | 1.5 | **281.0** | 2.5 | -32 | 6.5 | 8.1 | 8.7 | 8.7±2.0 |
|  |  | 1.5 | 310.0 | 2.5 | -19 |  |  |  | 8.7±2.0 |
| STLAC | 386.2 [M+H]^+^ | 1.5 | **95.3** | 2.0 | -26 | 6.5 | 7.6 | 8.4 | 8.0±3.0 |
|  |  | 1.5 | 178.0 | 2.0 | -7 |  |  |  | 8.0±3.0 |
| Table S3: continued from previous page. | | | | | | | | | |
| Analyte | **Q1 mass**  **[*m*/*z*]** | **Q1 resolution** | **Q3 mass**  **[*m*/*z*]** | **Q3 resolution** | **CE [eV]** | **Wait [min]** | **RT analyte peak [min]** | **RT echo peak [min]** | **RT set [min]** |
| STLAC AC | 428.2 [M+H]^+^ | 0.7 | **178.1** | 2.5 | -24 | 6.5 | 8.0 | 8.8 | 8.6±2.0 |
|  |  | 0.7 | 368.1 | 2.5 | -8 |  |  |  | 8.6±2.0 |

**Table S4:** Indoor building material samples showing quantifiable concentrations of the analyzed mycotoxins determined by UHPLC-TQMS, signal suppression and enhancement [SSE (%)] values determined by echo peak experiments and classification if a correction of the quantitative results was applied. Respective mycotoxin abbreviations are listed in Table S1.

| **Sample** | **Quantifiable mycotoxins** | **SSE echo peak [%]** | **Correction of quantitative results** |
| --- | --- | --- | --- |
| 2 | ACDIAL AC | 40.2 | yes |
|  | L-671 | 100.9 | no |
|  | SAT G | 105.6 | no |
|  | SAT H | 120.9 | no |
|  | STAM | 115.3 | no |
|  | STBON D | 85.9 | no |
|  | ST B | 113.8 | no |
|  | ST C | 125.7 | no |
|  | STDIAL | 56.6 | yes |
|  | STDIAL AC | 60.5 | yes |
|  | STLAC | 132.7 | yes |
|  | STLAC AC | 88.6 | no |
| 5 | ACDIAL AC | 83.2 | no |
|  | L-671 | 97.6 | no |
|  | STBON D | 91.8 | no |
|  | ST B | 94.0 | no |
|  | ST C | 90.3 | no |
|  | STDIAL | 72.9 | no |
|  | STDIAL AC | 80.7 | no |
|  | STG | 98.1 | no |
| 6 | ACDIAL AC | 67.8 | yes |
|  | L-671 | 102.5 | no |
|  | STBON D | 98.6 | no |
|  | ST B | 98.9 | no |
|  | ST C | 96.9 | no |
|  | STDIAL | 75.7 | no |
|  | STDIAL AC | 72.5 | no |
|  | STG | 107.9 | no |
|  | STLAC | 100.4 | no |
|  | STLAC AC | 95.7 | no |
| 7 | ACDIAL AC | 91.2 | no |
|  | L-671 | 94.8 | no |
|  | STAM | 112.1 | no |
|  | STBON D | 77.3 | no |
|  | ST B | 93.1 | no |
|  | ST C | 112.2 | no |
|  | STG | 94.7 | no |
|  | STDIAL | 106.9 | no |
|  | STDIAL AC | 87.8 | no |
|  | STLAC | 93.3 | no |
|  | STLAC AC | 83.7 | no |
| 8 | ACDIAL AC | 69.9 | yes |
|  | L-671 | 107.3 | no |
|  | STAM | 95.3 | no |
|  | STBON D | 99.7 | no |
|  | ST B | 96.9 | no |
|  | ST C | 78.6 | no |
|  | STDIAL | 83.4 | no |
|  | STDIAL AC | 61.3 | yes |
|  | STG | 112.5 | no |
|  | STLAC | 86.9 | no |
|  | STLAC AC | 98.2 | no |
| 9 | STG | 60.3 | yes |
| 10 | ENB | 79.9 | no |
|  | STG | 65.3 | yes |
| Table S4: continued from previous page. | | | |
| **Sample** | **Quantifiable mycotoxins** | **SSE echo peak [%]** | **Correction of quantitative results** |
| 12 | ENB | 96.4 | no |
|  | STG | 102.8 | no |
| 14 | STG | 103.6 | no |
| 15 | STG | 101.4 | no |
| 16 | STG | 98.2 | no |
| 17 | STG | 90.4 | no |
| 18 | STG | 107.4 | no |
| 19 | STG | 97.6 | no |
| 21 | ACDIAL AC | 23.4 | yes |
|  | L-671 | 62.5 | yes |
|  | STBON D | 42.2 | yes |
|  | ST C | 47.5 | yes |
|  | STDIAL | 59.2 | yes |
| 24 | ENB | 86.8 | no |
| 25 | STG | 112.5 | no |
| 26 | L-671 | 111.6 | no |
|  | STBON D | 98.1 | no |
| 29 | STG | 113.6 | no |
| 30 | STG | 129.1 | no |
| 31 | STG | 117.0 | no |
| 33 | STG | 130.0 | no |
| 36 | STG | 119.6 | no |
| 44 | STG | 104.7 | no |
| 49 | STG | 156.5 | yes |

**Table S5:** Detailed list of determined mycotoxin concentrations (including standard deviations) in analyzed indoor building material samples determined by UHPLC-TQMS. Each sample was analyzed in duplicate. Respective mycotoxin abbreviations are listed in Table S1.

| Sample | ACDIAL AC | ENA_1_ | ENB | | L-671 | SAT G | SAT H | STAM | STBON D | ST B | ST C | STCHR B | STDIAL | STDIAL AC | STG | STLAC | STLAC AC |
| --- | --- | --- | --- | --- | --- | --- | --- | --- | --- | --- | --- | --- | --- | --- | --- | --- | --- |
|  | [ng/cm^2^] | | | | | | | | | | | | | | | | |
| 2^*^ | 7740 (202) | n.d. | n.d. | 161  (11) | | 1381 (69) | 1267 (125) | 164  (11) | 691  (35) | 153  (29) | 76.9 (17.6) | <LOQ  (-) | 1468 (295) | 2377  (20) | n.d. | 3863 (378) | 218  (11) |
| 5 | 145  (7) | n.d. | n.d. | 17.1 (0.3) | | n.d. | n.d. | <LOQ (-) | 16.3 (2.6) | 29.6 (0.6) | 10.9 (0.1) | n.d. | 38.0  (-) | 77.0  (7.3) | 0.914 (0.239) | <LOQ (-) | <LOQ (-) |
| 6^*^ | 231  (6) | n.d. | n.d. | 18.1 (0.1) | | n.d. | n.d. | <LOQ (-) | 20.8 (1.9) | 31.1 (0.5) | 11.2 (0.0) | n.d. | 38.8  (0.3) | 92.6  (4.0) | 1.22 (0.06) | 281  (8) | 27.3  (-) |
| 7 | 586  (286) | n.d. | n.d. | 76.7 (44.1) | | n.d. | n.d. | 9.93  (-) | 92.4 (25.7) | 89.2 (43.2) | 23.9 (9.2) | n.d. | 139  (81) | 438  (155) | 2.23 (0.21) | 775 (372) | 85.0 (43.1) |
| 8^*^ | 343  (140) | n.d. | n.d. | 25.2 (7.4) | | n.d. | n.d. | 6.88  (-) | 48.3 (31.8) | 39.3 (9.3) | 12.9 (1.9) | n.d. | 70.7  (-) | 313  (189) | 0.749 (0.186) | 397  (-) | 38.5  (-) |
| 9^*^ | n.d. | n.d. | n.d. | n.d. | | n.d. | n.d. | n.d. | n.d. | n.d. | n.d. | n.d. | n.d | n.d | 979  (-) | n.d | n.d |
| 10^*^ | n.d. | n.d. | 0.369 (0.024) | n.d. | | n.d. | n.d. | n.d. | n.d. | n.d. | n.d. | n.d. | n.d | n.d | 0.886  (-) | n.d | n.d |
| 12 | n.d. | n.d. | 0.365 (0.025) | n.d. | | n.d. | n.d. | n.d. | n.d. | n.d. | n.d. | n.d. | n.d | n.d | 0.911  (-) | n.d | n.d |
| 14 | n.d. | n.d. | n.d. | n.d. | | n.d. | n.d. | n.d. | n.d. | n.d. | n.d. | n.d. | n.d | n.d | 0.571  (-) | n.d | n.d |
| 15 | n.d. | n.d. | n.d. | n.d. | | n.d. | n.d. | n.d. | n.d. | n.d. | n.d. | n.d. | n.d | n.d | 2.99 (0.10) | n.d | n.d |
| 16 | n.d. | n.d. | n.d. | n.d. | | n.d. | n.d. | n.d. | n.d. | n.d. | n.d. | n.d. | n.d | n.d | 0.830 (0.019) | n.d | n.d |
| 17 | n.d. | n.d. | n.d. | n.d. | | n.d. | n.d. | n.d. | n.d. | n.d. | n.d. | n.d. | n.d | n.d | 0.680 (0.034) | n.d | n.d |
| 18 | n.d. | n.d. | n.d. | n.d. | | n.d. | n.d. | n.d. | n.d. | n.d. | n.d. | n.d. | n.d | n.d | 0.952  (-) | n.d | n.d |
| 19 | n.d. | n.d. | n.d. | n.d. | | n.d. | n.d. | n.d. | n.d. | n.d. | n.d. | n.d. | n.d | n.d | 0.524  (-) | n.d | n.d |
| 21^*^ | 546  (-) | n.d. | n.d. | 25.7 (1.4) | | n.d. | n.d. | n.d. | 20.7 (0.0) | n.d. | 22.8  (-) | n.d. | 60.4  (0.4) | n.d | n.d. | n.d | n.d |
| Table S5: continued from previous page. | | | | | | | | | | | | | | | | | |
| Sample | **ACDIAL AC** | **ENA_1_** | **ENB** | **L-671** | | **SAT G** | **SAT H** | **STAM** | **STBON D** | **ST B** | **ST C** | **STCHR B** | **STDIAL** | **STDIAL AC** | **STG** | **STLAC** | **STLAC AC** |
|  | [ng/cm^2^] | | | | | | | | | | | | | | | | |
| 24 | n.d. | n.d. | 0.323 (0.000) | n.d. | | n.d. | n.d. | n.d. | n.d. | n.d. | n.d. | n.d. | n.d | n.d | n.d. | n.d | n.d |
| 25 | n.d. | n.d. | n.d. | n.d. | | n.d. | n.d. | n.d. | n.d. | n.d. | n.d. | n.d. | n.d | n.d | 0.813 (0.067) | n.d | n.d |
| 26 | n.d. | n.d. | n.d. | 16.3 (0.0) | | n.d. | n.d. | n.d. | 8.48 (0.04) | n.d. | n.d. | n.d. | n.d | n.d | n.d. | n.d | n.d |
| 29 | n.d. | n.d. | n.d. | n.d. | | n.d. | n.d. | n.d. | n.d. | n.d. | n.d. | n.d. | n.d | n.d | 3.67  (-) | n.d | n.d |
| 30 | n.d. | n.d. | n.d. | n.d. | | n.d. | n.d. | n.d. | n.d. | n.d. | n.d. | n.d. | n.d | n.d | 9.91  (-) | n.d | n.d |
| 31 | n.d. | n.d. | n.d. | n.d. | | n.d. | n.d. | n.d. | n.d. | n.d. | n.d. | n.d. | n.d | n.d | 0.516  (-) | n.d | n.d |
| 33 | n.d. | n.d. | n.d. | n.d. | | n.d. | n.d. | n.d. | n.d. | n.d. | n.d. | n.d. | n.d | n.d | 0.992 (0.215) | n.d | n.d |
| 36 | n.d. | n.d. | n.d. | n.d. | | n.d. | n.d. | n.d. | n.d. | n.d. | n.d. | n.d. | n.d | n.d | 1.26 (0.70) | n.d | n.d |
| 44 | n.d. | n.d. | n.d. | n.d. | | n.d. | n.d. | n.d. | n.d. | n.d. | n.d. | n.d. | n.d | n.d | 0.928 (0.317) | n.d | n.d |
| 45 | n.d. | <LOQ  (-) | <LOQ (-) | n.d. | | n.d. | n.d. | n.d. | <LOQ (-) | n.d. | n.d. | n.d. | n.d | n.d | <LOQ (-) | n.d | n.d |
| 46 | n.d. | n.d. | n.d. | n.d. | | n.d. | n.d. | n.d. | n.d. | n.d. | n.d. | n.d. | n.d. | n.d. | <LOQ (-) | n.d. | n.d. |
| 49^*^ | n.d. | n.d. | n.d. | n.d. | | n.d. | n.d. | n.d. | n.d. | n.d. | n.d. | n.d. | n.d | n.d | 3.33 (1.33) | n.d | n.d |
| 51 | n.d. | n.d. | <LOQ (-) | n.d. | | n.d. | n.d. | n.d. | n.d. | n.d. | n.d. | n.d. | n.d | n.d | n.d. | n.d | n.d |

^*^ Echo correction was performed.

n.d. Not detectable.

- Not determinable.
